# Baculovirus-Displayed ASFV Epitope-Composite Protein Elicits Potent Immune Responses

**DOI:** 10.3390/microorganisms13112468

**Published:** 2025-10-29

**Authors:** Wenkai Zhang, Xing Yang, Xingyu Chen, Jiaxin Jin, Yuanyuan Zhang, Lele Gong, Shuai Zhang, Xuyang Zhao, Yongkun Du, Yanan Wu, Aijun Sun, Guoqing Zhuang

**Affiliations:** 1 International Joint Research Center of National Animal Immunology, College of Veterinary Medicine, Henan Agricultural University, Zhengzhou 450046, China; 2 Longhu Laboratory of Advanced Immunology, Zhengzhou 450046, China

**Keywords:** ASFV, recombinant protein, baculovirus, vaccine

## Abstract

African swine fever (ASF), caused by the African swine fever virus (ASFV), is an acute, febrile, highly contagious, and lethal disease that poses a severe threat to the global pig farming industry. Currently, no globally recognized, safe, and effective commercial ASF vaccine has been developed, making vaccination a crucial strategy for outbreak control. The ASFV structural proteins p72, p30, and p54 are key targets for vaccine development. In this study, we developed a novel baculovirus vector-based system for surface display of a recombinant protein comprising epitopes from p72, p30, and p54. Upon infection, the recombinant protein was expressed and anchored on the plasma membrane of Sf-9 cells. Purified virus analysis revealed that the Bac-recombinant protein enhanced gene delivery and transgene expression in mammalian cells compared to the Bac-Wild Type (Bac-WT). In a murine model, the Bac-recombinant protein induced significantly higher IFN-γ and IL-4 levels than Bac-p30 and the negative control. However, further evaluation in swine models is required to confirm its protective potential against ASFV. Furthermore, it also elicited a robust antibody response, generating high-titer Bac-recombinant protein-specific antibodies. Therefore, these findings suggest that the ASFV Bac-recombinant protein is a promising candidate for a vector-based vaccine.

## 1. Introduction

Since the first report of African swine fever virus (ASFV) in Kenya in 1921, the virus has spread to over 80 countries across four continents [1]. Between 2022 and August 2024, ASFV caused the loss of more than 1,727,000 animals, resulting in severe economic losses for the global pig breeding industry (World Organization for Animal Health [WOAH], accessed on 8 November 2024). In China, ASFV is classified as a first-class animal epidemic requiring key preventive measures [2]. The ASFV genome, ranging from 170 to 193 kb, includes 24 distinct genotypes based on the B646L (p72) gene, while only a limited number of serogroups have been proposed without a standardized serotype classification [3]. ASFV-encoded structural proteins, including pp220, pp62, p72, p54, p30, and CD2v, are essential for viral particle formation, mediating viral attachment, entry, and replication. Among these, p72 is a key component of the viral capsid, playing a critical role in its assembly during the late stages of infection. It is the major constituent of the viral icosahedron and exhibits strong antigenicity and immunogenicity [4]. The *E183L* gene encodes p54, a major structural protein essential for ASFV replication and characterized as a viral attachment protein. p54 also activates effector caspase-3, inducing apoptosis in the early stages of ASFV infection [5]. The ASFV p30 protein, encoded by the *CP204L* gene, has a relative molecular weight of ~30 kDa and appears on the cell membrane shortly after infection. It may regulate signal transduction and is crucial for viral internalization into host cells [6].

Since the emergence of ASFV, it has drawn significant attention in virology and veterinary medicine, both domestically and internationally. Efforts to develop effective vaccines against this viral disease have been ongoing [7,8,9,10]. Current ASF vaccine development primarily focuses on attenuated vaccines with gene deletions and subunit vaccines containing protective antigens. Live-attenuated vaccines are the primary focus for ASF but carry biosafety concerns. In contrast, protein-based vaccines offer a superior safety profile but have yet to demonstrate comparable efficacy, highlighting a critical challenge for the field. Studies have shown that the highly immunogenic proteins p30, p54, and p72 induce neutralizing antibodies against ASFV. Specifically, p30 antibodies prevent virus internalization, while p54 and p72 antibodies inhibit virus adsorption [11]. Immunization with a combination of p72, p54, p30, and p22 proteins can delay symptom onset [12]. However, protein-based vaccines require adjuvants to enhance immunogenicity, and antigen hydrophobicity complicates purification, reducing cost-effectiveness. DNA vaccines use genetically engineered DNA to induce humoral and cell-mediated immune responses against parasites, bacteria, and viruses [13]. However, concerns remain that DNA vaccines may trigger anti-DNA antibody production, potentially leading to autoimmune responses and immune tolerance in the host.

Live-vector vaccines, a type of genetically engineered vaccine, use non-pathogenic viruses or bacteria as carriers to express protective antigen genes. Upon infection, these vectors enable target antigen expression, leading to endogenous antigen processing and MHC class I-restricted antigen presentation [14]. Baculoviruses, double-stranded DNA viruses that infect invertebrates, are widely employed for vaccine gene expression in insect cells both in vitro and in vivo. Among them, Autographa californica multicapsid nucleopolyhedrovirus (AcMNPV) is the most extensively studied and has been used to overexpress recombinant proteins in insect cells [15]. When driven by the CMV promoter, modified AcMNPV can also express foreign genes in mammalian cells. Due to their ability to achieve high exogenous protein expression and post-translational modifications, baculoviruses are considered promising vectors for gene therapy and vaccine development [15,16,17]. To enhance baculovirus transduction efficiency, vesicular stomatitis virus G protein (VSV-G) or GP64 signal peptide is often used to facilitate surface presentation of target proteins. VSVG-pseudotyped recombinant baculoviruses have been used to express H5N1 avian influenza virus HA [18], rabies virus G protein [19], porcine circovirus type 2 Cap protein, and infectious bronchitis virus S1 protein [20]. These studies suggest that baculoviruses hold potential as vaccine vectors for clinical applications [21]. However, research on recombinant baculoviruses as immunizing agents for inducing protective immunity against viral infections remains limited in the ASFV research. Since p72, p54, and p30 are key immunogens eliciting anti-ASFV responses and pseudotyped baculoviruses are promising vaccine vectors, this study aimed to efficiently display recombinant proteins on the baculovirus envelope, ensuring their strong immunogenicity during in vivo immune responses.

This study will construct a recombinant baculovirus encoding a fusion protein that incorporates linear epitopes from p72, p54, and p30. The protein will be displayed on a defined protein scaffold and tagged with a 6×His epitope. Additionally, a porcine Fc fragment was co-expressed to enhance the stability and persistence of the recombinant protein in vivo. The recombinant CMV-promoter-driven baculovirus will first be assessed for efficient transduction in mammalian cells. Subsequently, the immunogenic potential of the purified virus particles will be evaluated in vivo.

## 2. Materials and Methods

### 2.1. Experimental Materials

Plasmids pFastBacDual and DH10Bac Chemically Competent Cells were purchased from Beijing Zoman Biotechnology Co., Ltd., Beijing, China. The p72 monoclonal antibody (mAb), p30 mAb, BHK cells, PK-15 cells, and Sf9 cells were maintained in our laboratory. ASF-positive serum was obtained from the China Veterinary Drug Supervision Institute (Beijing, China). SPF BALB/c mice were purchased from Beijing Vital River Laboratory Animal Technology Co., Ltd. (Beijing, China). Baculovirus (AcMNPV) and Sf9 cells were cultured in SIM SF (SinoBiological, Houston, TX, USA) serum-free medium at 27 °C, supplemented with 100 μg/mL penicillin and 100 μg/mL streptomycin. Recombinant viruses were propagated in Sf9 cells. Complete and incomplete Freund’s adjuvants (Sigma, St. Louis, MO, USA) were used for mouse immunization.

### 2.2. Optimization and Screening of ASFV Multi-Epitope Combination Peptides

In our previous study, the linear epitopes of p54 and p30 proteins were predicted using tools from the Immunomedicine Group (http://imath.med.ucm.es/bepiblast/, accessed on 8 September 2022) and BepiPred 2.0 (http://tools.iedb.org/bcell/result/, accessed on 8 September 2022) based on hydrophobicity analysis. Antigenic epitopes exposed on the protein surface were selected based on hydrophilicity, antigenicity, and conservation analyses, while the p72 epitopes were identified in our laboratory [22]. A structurally stable and solubly expressed backbone protein was chosen as a scaffold for antigenic epitope peptides [23]. The spatial structure of antigenic epitope polypeptide recombinant proteins was predicted using AlphaFold-2 v2.1.1 online software, confirming that the original spatial structure of the proteins remained unchanged.

### 2.3. Design and Construction of Recombinant Baculovirus Structures

The commercial pFastBacDual baculovirus expression system shuttle plasmid was modified to enhance its applicability in our research. Our team has engineered a derivative plasmid, pFastBacDual-CMV-GP64SP-FC-VSVG, containing an expression cassette regulated by the polyhedron (polh) promoter. This cassette includes the GP64-SP signal peptide, porcine IgG Fc protein, VSV-G protein, and the SV40 poly(A) tail. Furthermore, CMV promoters and enhancers were incorporated to optimize eukaryotic expression in mammalian cells. The stability of this expression system was assessed [24]. The pFastBacDual-CMV-GP64SP-FC-VSVG plasmid, recombinant protein, and p30 gene sequences (ASFV pig/HLJ/2018 strain, GenBank ID: IDMK333180.1) were optimized and synthesized by Shanghai Sangon Bioengineering Co., Ltd. (Shanghai, China). Primers were designed to amplify the full-length recombinant protein and p30 sequences using the synthetic gene as a template (Table 1). The PCR product and pFastBacDual vector were digested with *Kpn*I and *Xho*I, then ligated with T4 ligase at 16 °C. The recombinant plasmid was transformed into TOP10 competent cells, which were grown on ampicillin-containing LB agar at 37 °C. Positive colonies were selected, and plasmids were extracted. Recombinant plasmids were then transformed into DH10Bac-competent cells and plated on LB agar containing IPTG, X-Gal, tetracycline, kanamycin, and gentamicin. Positive and negative colonies were distinguished via blue-white screening. Recombinant bacmid DNA was extracted and transfected into Sf9 cells to generate recombinant baculovirus. The recombinant protein and p30 protein produced by the baculovirus expression system are denoted by ‘Bac-RP’ and ‘Bac-p30’. After two passages, virus titers were determined using the endpoint dilution method [25]. Recombinant protein expression was analyzed in fourth-generation virus cultures. The cell supernatants were collected and stored at −80 °C.

### 2.4. Western Blot

The expression of Bac-RP and Bac-p30 was confirmed by Western blot analysis. Infected cell lysates were subjected to 12.5% sodium dodecyl sulfate-polyacrylamide gel electrophoresis (SDS-PAGE) and transferred onto nitrocellulose membranes. The membranes were blocked at room temperature (RT) for 1 h with 5% skim milk, followed by incubation with anti-His antibodies (1:2000), p30 monoclonal antibody (mAb) (1:2000), or ASFV-positive serum (1:1000) for 2 h at RT. After five washes with phosphate-buffered saline containing 0.01% Tween-20 (PBST), the membranes were incubated for 1 h at RT with HRP-conjugated goat anti-mouse IgG (ABclonal Technology Co., Ltd., Wuhan, China) or HRP-conjugated goat anti-swine IgG (Abcam, Cambridge, MA, USA) at 1:3000 and 1:5000 dilutions, respectively. The negative control cells were processed identically. After five additional PBST washes, hypersensitive chemiluminescence reagent (Enhanced Chemiluminescence, ECL) (New Cell & Molecular Biotech Co., Ltd., Suzhou, China) was applied, and target proteins were visualized using the Amersham Imager 680 (GE Co., Ltd., Boston, MA, USA) with an exposure time of 2 min.

### 2.5. Immunofluorescence Assay

Sf9 cells were cultured on sterile coverslips placed in 12-well plates and infected at a multiplicity of infection (MOI) of 10. After 72 h of infection, the culture medium was removed, and the cells were fixed with 4% paraformaldehyde. Permeabilization was performed using 0.1% Triton X-100 (Solarbio, Beijing, China). After three washes with PBS (pH 7.4), cells were blocked with 5% skim milk at 37 °C for 1 h, followed by washing thrice and incubating cells with primary antibody (p72/p30 mAb, 1:300) for 1 h at 37 °C. After three PBS washes, cells were incubated with FITC-conjugated goat anti-mouse or goat anti-pig mAb (1:200) for 1 h at 37 °C, followed by three additional PBS washes. Negative control cells were treated identically. Cellular DNA was stained with 4ʹ,6-diamidino-2-phenylindole (DAPI, Beyotime, Shanghai, China). Fluorescence microscope (Olympus Corporation, Tokyo, Japan) and an LSM 800 laser-scanning confocal microscope (Zeiss, Oberkochen, Germany) were used for imaging.

### 2.6. Transduction of Mammalian Cells by the Recombinant Baculovirus

BHK or PK-15 cells were seeded into 24-well plates and cultured until they reached approximately 70–80% confluence. After removing the medium, the cells were washed three times with PBS (pH 7.4). Baculovirus (MOI = 20) was added and incubated with the cells for 1 h at room temperature (22–25 °C) to allow viral adsorption. Following viral removal, fresh medium was added, and the cultures were incubated at 37 °C to permit gene expression. At 48 h post-transduction, cells were harvested for RNA extraction. RT-qPCR was performed to assess the transduction of recombinant proteins in mammalian cells. The primers used are listed in Table 1.

### 2.7. Virus Purification

Recombinant baculoviruses were produced by infecting Sf9 cells at an MOI of 10 and harvested 4 days post-infection. For large-scale production, viral supernatants were purified via ultracentrifugation through two rounds of sucrose gradient separation (20–60%) following a standard protocol. The purified recombinant baculovirus was analyzed by Western blot using p72 and p30 monoclonal antibodies. Viral titers were determined by fluorescence quantification [26].

### 2.8. Mouse Immunization

Female BALB/c mice (6–8 weeks old) were randomly assigned to four groups: PBS negative control, Bac-WT, Bac-RP, and Bac-p30 (*n* = 5 per group). Mice were immunized three times via subcutaneous injection on the back. Freund’s complete adjuvant was used for the primary immunization, while Freund’s incomplete adjuvant was used for booster doses. Each mouse received 1 × 10^8^ PFU of recombinant baculovirus. Immunized mice were housed in a specific pathogen-free (SPF) facility at Henan Agricultural University. Two weeks after the third immunization, serum samples were collected from the retroorbital plexus for serological analysis and antibody quantification. Mice were euthanized, and all experimental procedures were conducted in strict accordance with the Guide for the Care and Use of Laboratory Animals. Splenocytes were isolated for cell subtyping analysis.

### 2.9. Detection of Cytokines

To evaluate cellular immunity, IL-4, IL-6, IFN-α, and IFN-γ levels in mouse serum from all experimental groups were measured two weeks after the third immunization using a commercial cytokine assay kit (Shanghai Enzyme-Linked Biotechnology Co., Ltd., Shanghai, China) following the manufacturer’s instructions.

### 2.10. Detection of T Cell Subtypes

Mouse spleens were collected two weeks after the third immunization. Spleen lymphocytes (2 × 10^6^ cells/mL) were isolated using a lymphocyte separation medium, washed twice with PBS, and incubated with BSA at room temperature for 30 min. Cells were then stained with PE-labeled CD3, APC-A700-labeled CD4, and FITC-labeled CD8 (Proteintech, Wuhan, China) and incubated at 4 °C in the dark for 30 min. After two PBS washes, fluorescence analysis was performed using flow cytometry (Becton Dickinson FACS Calibur system, Franklin Lakes, NJ, USA) to assess CD3, CD4, and CD8 expression. A minimum of 10,000 events per sample were collected and analyzed using CytExpert software 2.4.

### 2.11. Statistical Analysis

Statistical analyses were conducted using GraphPad Prism 9 (GraphPad Software Inc., San Diego, CA, USA). Data are presented as mean ± standard deviation (SD). Unpaired *t*-tests or two-way analysis of variance (ANOVA) were used for statistical analyses, with significance set at *p* < 0.05.

## 3. Results

### 3.1. Construction of the Recombinant Baculoviruses

**Figure 1:**
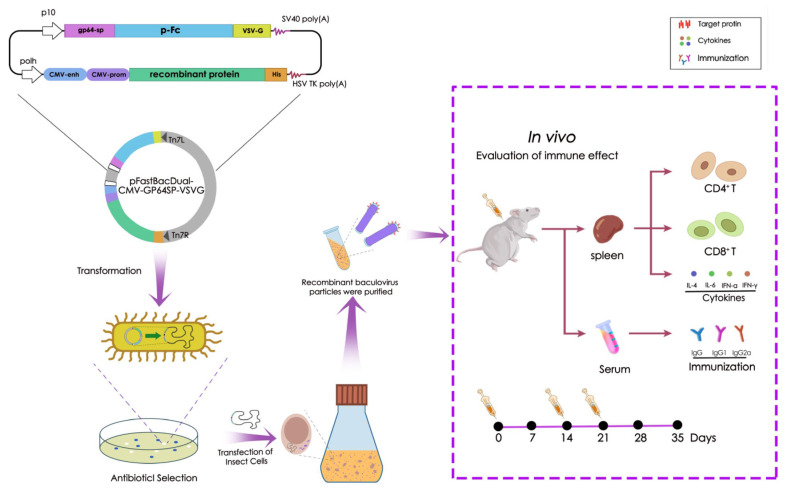
Overview of the process and application of recombinant baculoviruses generated using the Bac-to-Bac Baculovirus Expression System. The constructed recombinant baculovirus vector, pFastBacDual-GP64SP-FC-VSVG, is illustrated. The target proteins were cloned into a baculovirus vector containing a fusion gene fragment comprising the gp64 SP signal peptide, porcine-derived IgG Fc fragment (pFc), vesicular stomatitis virus G protein (VSV-G), and human cytomegalovirus (CMV) enhancer/promoter cassette. Notably, Bac-RP induces elevated cytokine secretion, IgG titers, and CD4+ and CD8+ T cell activation while maintaining high biosafety in murine models.

As previously stated, the recombinant protein gene (Bac-RP/Bac-p30) is regulated by a dual promoter system consisting of the p10 (P10) and CMV promoters. Furthermore, the baculovirus signal peptide GP64-SP, the porcine IgG Fc fragment (pFc), and the VSV-G protein are concatenated under the control of the polh promoter, with their sequences confirmed by sequencing analysis. During budding, baculovirus acquires the host cell membrane as its envelope. GP64-SP facilitates protein translocation to the insect cell plasma membrane, exposing it to the extracellular surface. The efficiency of target protein display on the host cell membrane directly reflects its display efficiency on virus particles (Figure 1).

### 3.2. Identification of Recombinant Protein

**Figure 2:**
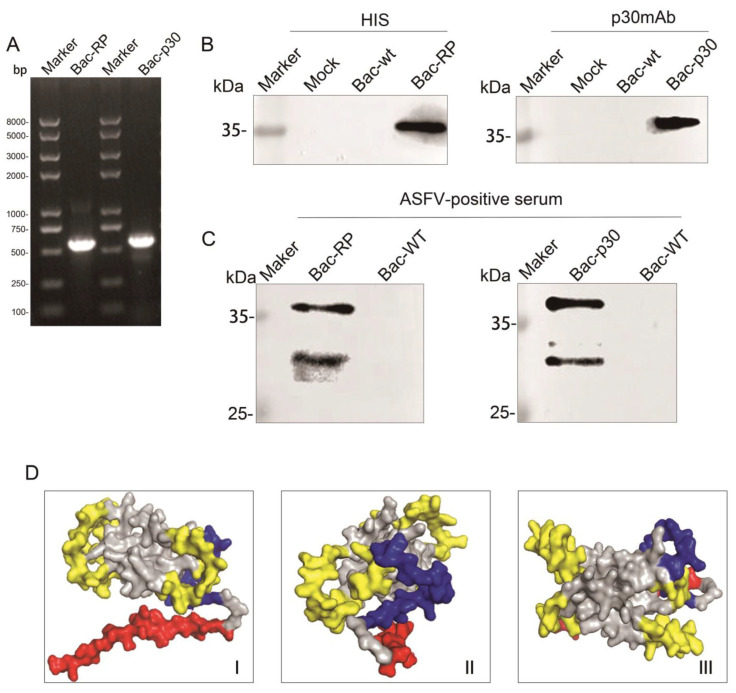
Expression of Bac-RP and Bac-p30 in Sf9 cells. (**A**) PCR amplification of the recombinant protein/p30 gene. (**B**) Western blot analysis using anti-His and p30 monoclonal antibodies to confirm Bac-RP and Bac-p30 expression. (**C**) Western blot analysis using ASFV-positive serum as the primary antibody to verify the identity of the purified Bac-RP and Bac-p30. (**D**) Structural prediction of Bac-RP using PyMOL 2.4.1. Epitopes recognized by p72, p54, and p30 are highlighted in yellow, blue, and red, respectively. The structure is displayed from (I) frontal, (II) lateral, and (III) top perspectives.

To obtain Bac-RP and Bac-p30, the corresponding gene fragments were cloned into the pFastBacDual-CMV-GP64SP-FC-VSVG shuttle plasmid and integrated into the Bac-to-Bac bacmid via homologous recombination. Sf9 cells were transfected to propagate the virus, and viral particles were collected after multiple passages. The MOI was set at 10. The *recombinant protein* and *p30* gene fragments were amplified by PCR (Figure 2A). Western blot analysis (Figure 2B) showed that cells infected with Bac-WT did not express proteins detectable by anti-His or anti-p30 antibodies. In contrast, cells infected with Bac-RP or Bac-p30 expressed a ~35 kDa protein detected by both antibodies, confirming the expression of recombinant protein and p30. Further Western blot analysis using ASFV-positive serum as the primary antibody identified two distinct bands at ~35 kDa and ~27 kDa (Figure 2C), corresponding to the target protein and the pFc protein expressed under the polh promoter. In contrast, Bac-WT-infected cells did not produce proteins recognizable by ASFV-positive serum antibodies. The spatial structure of the antigenic epitope polypeptide recombinant protein was visualized using PyMOL 2.4.1 (Figure 2D). Different colors represent distinct protein epitopes, all of which are exposed on the molecular surface.

### 3.3. Cell Location of Recombinant Proteins

**Figure 3:**
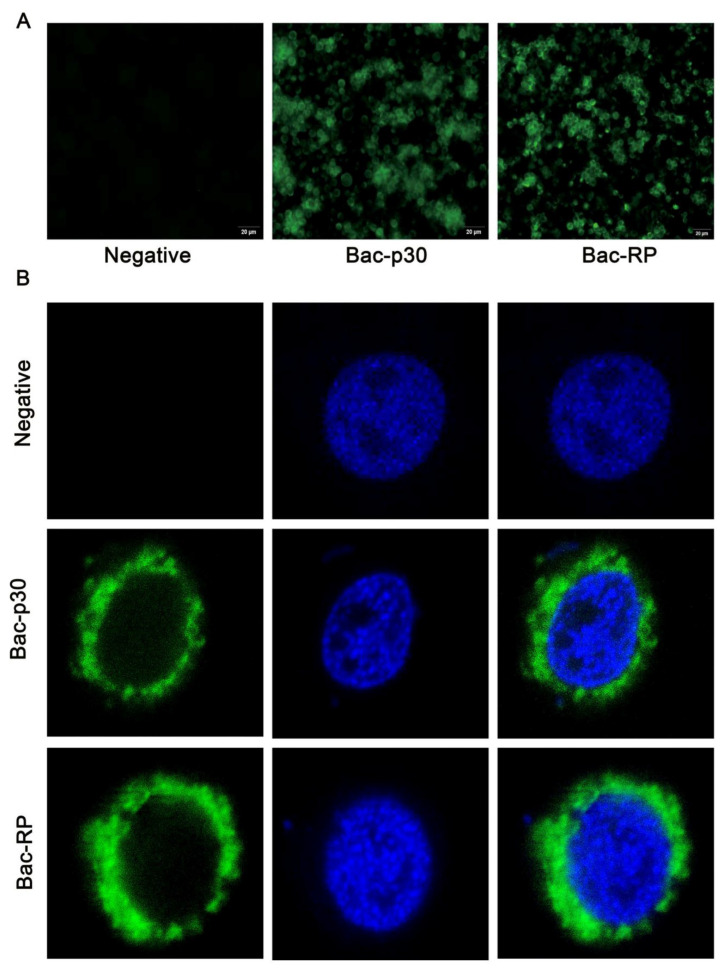
Confocal laser scanning microscopy images of Sf9 cells incubated with FITC-labeled (green) Bac-RP/Bac-p30. Cells were incubated with primary antibodies and FITC-conjugated secondary antibodies, with nuclei counterstained using DAPI (blue). (**A**) Specificity analysis of Bac-RP/Bac-p30 by IFA assay. (**B**) Localization of Bac-RP/Bac-p30 in Sf9 cells.

To assess whether Bac-RP and Bac-p30 are correctly transferred to the cell surface, cells were cultured on sterile coverslips in 24-well plates and infected at an MOI of 10. As shown in Figure 3A,B, Bac-WT did not express detectable surface proteins when probed with anti-p72 or anti-p30 antibodies. In contrast, Bac-RP and Bac-p30 were detected using these antibodies and localized to the plasma membrane, confirming the anchoring of p30 and the recombinant protein on the Sf9 cell surface.

### 3.4. Transduction, Purification and Characterization of Recombinant Baculovirus

**Figure 4:**
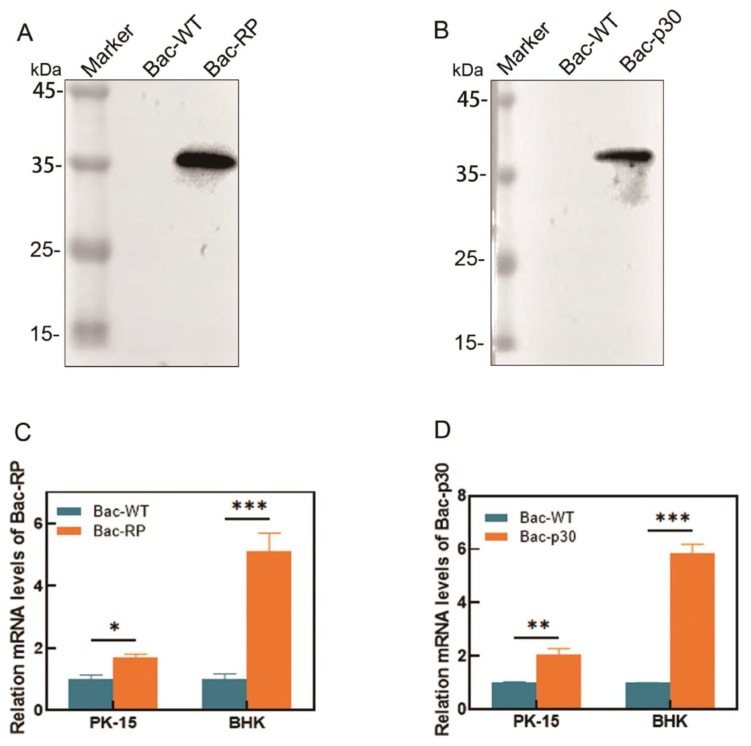
Characterization of Bac-RP/Bac-p30 expressed in baculovirus-infected Sf9 cells. (**A**,**B**) Ultracentrifugation of infected cell lysates on a 20–60% sucrose gradient. Bac-WT was used as a control. Western blot analysis using p72/p30 monoclonal antibodies confirmed the identity of the purified Bac-RP/Bac-p30. (**C**,**D**) PK-15 and BHK cells were inoculated with Bac-RP and Bac-p30 (MOI = 20). At 48 h post-inoculation, total RNA was extracted, and mRNA expression of Bac-RP and Bac-p30 was quantified by real-time PCR. Data were normalized to actin mRNA levels. *, *p* < 0.05; **, *p* < 0.01; ***, *p* < 0.001.

The recombinant protein was identified by Western blot and subsequently purified. To enhance purity, lysates of infected Sf9 cells underwent enrichment via 20–60% sucrose gradient ultracentrifugation. After two rounds of purification, the expression of Bac-RP (Figure 4A) and Bac-p30 (Figure 4B) was confirmed by Western blot, with both proteins exhibiting the expected sizes. To preliminarily assess CMV promoter-driven transgene expression in mammalian cells, BHK and PK-15 cells were transduced with recombinant baculoviruses at an MOI of 20. Weak and transient transcriptional signals were detected by qPCR (Figure 4C,D), consistent with non-replicative gene delivery by BacMam vectors. These results suggest potential for transient expression but require confirmation at the protein level.

### 3.5. The Recombinant Baculovirus Induced High Antibody Titer in Humoral Immune Response

**Figure 5:**
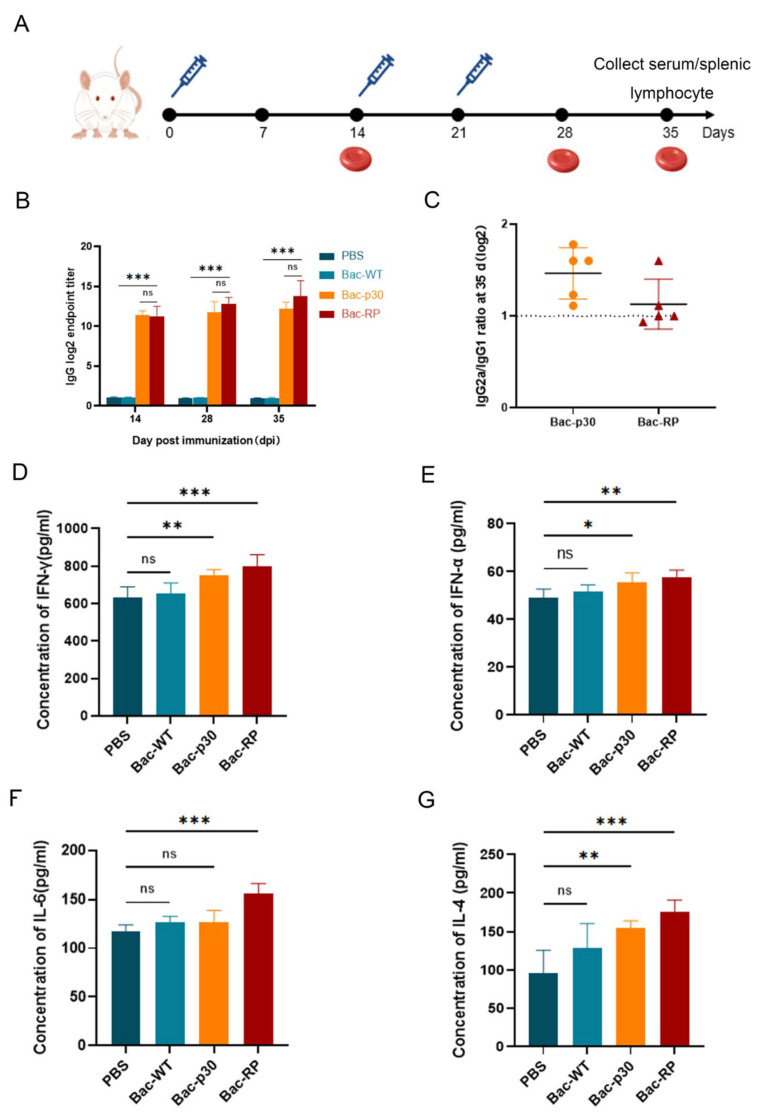
Immunization strategy and serum antibody/cytokine detection in BALB/c mice. (**A**) Experimental timeline for immunization and sample collection. Mice were immunized via intramuscular injection on days 0, 14, and 28. Serum was collected on days 14, 28, and 35, while spleen lymphocytes were harvested on day 35. (**B**) Detection of specific IgG antibodies in mouse serum. (**C**) Ratio of specific IgG2a to IgG1 in mouse serum at 35 days post-immunization, analyzed by ELISA. (**D**–**G**) Serum cytokine levels (IFN-γ, IFN-α, IL-6, IL-4) at 35 days post-immunization. PBS and Bac-WT groups were used as controls. *, *p* < 0.05; **, *p* < 0.01; ***, *p* < 0.001; ns = not significant (*p* > 0.05).

To evaluate whether recombinant baculovirus-expressed proteins induce specific humoral immune responses in vivo, mice were immunized via subcutaneous injection on the back with Bac-RP, Bac-p30, Bac-WT, or PBS. Bac-RP- and Bac-p30-specific ELISA antibodies were measured 14, 28, and 35 days after primary immunization (Figure 5A). Recombinant protein- and p30-specific antibody responses were detected in all mice vaccinated with Bac-RP and Bac-p30 by day 14 (Figure 5B). Following booster immunization, specific antibody levels increased rapidly. As expected, no specific antibodies were detected in the Bac-WT or PBS groups throughout the experiment. The differences were statistically significant (*p* < 0.05) (Figure 5B). These findings indicate that both Bac-RP and Bac-p30 are immunogenic in vivo and induce robust antibody production. To assess the relationship between the proportions of IgG1 and IgG2a in IgG produced by different immune groups, IgG1- and IgG2a-specific antibody titers were measured in serum on day 35. The IgG2a/IgG1 titer ratio is shown in Figure 5C. The immune response in mice immunized with Bac-RP and Bac-p30 was skewed toward a Th1-type cellular immune response.

### 3.6. Effects of Recombinant Proteins on Cytokines

One week after the final booster immunization, serum was collected from blood drawn from the caudal vein. A Mouse Cytokine ELISA Detection Kit was used to quantify IFN-γ, IFN-α, IL-4, and IL-6 levels (Figure 5D–G). Compared to the Bac-WT and PBS control groups, Bac-RP immunization significantly increased serum levels of IFN-γ, IFN-α, IL-4, and IL-6. These results suggest that Bac-RP promotes B and T lymphocyte proliferation and triggers an inflammatory response. Similarly, Bac-p30 immunization significantly increased IFN-γ, IFN-α, and IL-4 levels (Figure 5D,E,G), but IL-6 levels remained comparable to those in the Bac-WT control group (Figure 5F). Compared to Bac-WT and PBS groups, Bac-RP and Bac-p30 immunization increased serum cytokine levels.

### 3.7. Effect of Recombinant Proteins on Cellular Immune Response

**Figure 6:**
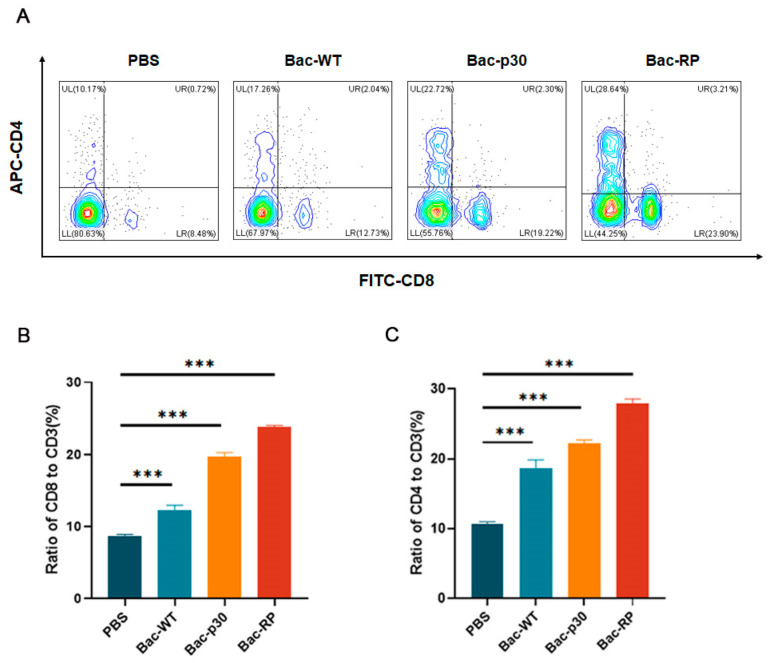
T cell responses induced by Bac-RP and Bac-p30. BALB/c mice (*n* = 3/group) were sacrificed on day 35 post-immunization, and splenic lymphocytes were isolated. (**A**) Quantification of CD8+ and CD4+ T cells by FACS. (**B**,**C**) Percentage of CD4+ and CD8+ T cells in splenic lymphocytes. Results are expressed as means ± SEM (*n* = 3). ***, *p* < 0.001.

To determine whether Bac-RP influences cellular immune responses, we evaluated T lymphocyte-mediated immunity. Lymphocytes were isolated from the spleens of mice 35 days post-immunization, and T cell subsets were analyzed by flow cytometry. Compared to the Bac-WT and PBS groups, Bac-RP significantly increased the proportion of activated CD4+ T lymphocytes to 28.64%, 1.6-fold higher than in the Bac-WT group (*p* < 0.001; Figure 6A). Similarly, Bac-RP elevated CD8+ T lymphocyte levels to 23.9%, 1.87-fold higher than in the Bac-WT group, with a statistically significant difference (*p* < 0.001; Figure 6A). Additionally, CD4+ and CD8+ T lymphocyte proportions were significantly higher in the Bac-p30-immunized group than in the control groups (PBS and Bac-WT) (Figure 6B,C). These findings indicate that Bac-RP enhances specific cellular immune activation.

## 4. Discussion

ASFV remains a major threat to the global pig industry, causing substantial economic losses due to its widespread transmission and impact. Effective vaccination is essential for controlling viral dissemination. To enhance immune responses and improve protection against ASFV, we evaluated a novel baculovirus-vectored vaccination strategy.

Compared to other viral vectors, baculovirus is considered both safe and efficient and has been widely used for various applications [19,27,28]. Vaccines displaying antigens on the baculovirus surface have demonstrated efficacy in eliciting protective immune responses in animal models [18,20]. By incorporating the GP64 structural domain (signal sequence, transmembrane, and cytoplasmic regions), foreign proteins such as human immunodeficiency virus (HIV) GP120, rubella virus envelope protein, and synthetic IgG binding domains can be displayed on the baculovirus envelope [29,30,31]. In avian influenza virus research, the baculovirus vectors have been engineered for HA expression using either the polh promoter (vAc-HA) or a dual polh-CMV promoter system (vAc-HA-DUAL). Vaccination with vAc-HA primarily elicited a Th2 immune response in mice, whereas vAc-HA-DUAL induced a Th1-biased response. Additionally, the vAc-HA-DUAL vaccine more effectively stimulated HA-specific CD4+ and CD8+ T cell responses in vitro [32]. In another study, Feng et al. displayed the full ectodomain of the SARS-CoV spike (S) protein on the baculovirus envelope using the GP64 signal peptide and VSV-G membrane anchor. In mice, this vaccine induced specific neutralizing antibodies against the S protein without adjuvants [33]. In the present study, we displayed IgG Fc protein using the GP64 signal peptide and VSV-G membrane anchor. The GP64-SP signal peptide enhances recombinant protein display on the cell surface, while the porcine IgG Fc fragment acts as a complement inhibitor, protecting recombinant proteins from serum complement-mediated inactivation [24,34]. Furthermore, the transmembrane domain (TMD) of VSV-G expands the host range and improves transduction efficiency in mammalian cells [35,36,37,38]. Simultaneously, the baculovirus vector was engineered with a dual polh-CMV promoter system to facilitate Bac-RP expression. Immunized mice exhibited a Th1-biased immune response, consistent with previous findings.

Early research on ASFV protein-based vaccines focused on developing antigens capable of inducing neutralizing antibody responses. The first recombinant ASFV protein shown to confer protection against ASFV challenge was the hemagglutinin protein CD2v, expressed via baculovirus [39]. Immunization with CD2v provided protection against a lethal challenge with the highly virulent ASFV IE75 strain, although viremia persisted [40]. Subsequent studies explored other ASFV structural proteins, including p12, p30, p54, and p72, assessing their protective potential as recombinant antigens. Research indicates that antibodies against p72 and p12 can hinder viral binding to host cells, while those targeting p30 can prevent viral entry [41,42]. However, p12-specific antibodies do not completely block virus binding or infectivity. The p30 and p54 proteins mediate ASFV-host cell interactions and elicit virus-neutralizing antibodies during infection. Most viruses possess multiple outer proteins that contribute to neutralization, complicating the process [43]. The immunoprotection afforded by currently available ASFV subunit vaccines remains inadequate; vaccinated animals still develop clinical signs and characteristic lesions following virulent challenge. Compared with subunit formulations, live-attenuated ASFV vaccines have demonstrated marked protective efficacy. The commercially licensed Vietnamese strains ASFV-G-ΔI177L and ASFV-G-ΔMGF elicit minimal adverse reactions and high protection rates, yet they fail to confer protection against newly emerged, highly virulent field isolates [44]. The widespread use of attenuated vaccines may inherently increase the risk of virulence reversion, precluding their large-scale global deployment on safety grounds. Efficient and safe control of ASFV is regarded as the paramount objective in vaccine development. Each protein typically contains multiple neutralization epitopes and may employ distinct neutralization mechanisms, with varying efficiency. In this study, recombinant proteins expressed via the baculovirus system comprising epitopes from p72, p54, and p30 were selected. These epitopes had been previously identified and validated [22]. Western blot analysis confirmed that the Bac-RP were recognized by anti-His and p72 monoclonal antibodies, demonstrating strong antigenicity.

The IgG Fc protein enhances antigen presentation and induces stronger immunity, making it highly promising for vaccine development [45]. Porcine FcRI receptors, widely expressed on antigen-presenting cells (APCs) such as dendritic cells and macrophages, are potential targets of porcine IgG Fc domains. The binding of porcine IgG Fc to FcRI receptors is hypothesized to enhance immune responses [46,47]. To promote antigen uptake by APCs without compromising linear epitope display, porcine IgG Fc proteins were co-expressed. The Fc region mediates potent immune effector functions via interactions with FcRI and serum complement proteins [48]. In our study, hybrid promoters significantly improved the expression (Figure 2C) and anchoring (Figure 3B) of fusion protein on recombinant baculovirus envelopes, enhancing immunogenicity. Western blot analysis using ASFV-positive serum confirmed the co-expression of Bac-recombinant and Fc proteins. These findings suggest that antigen presentation on the baculovirus surface may enhance APC phagocytic activity. APCs then process and either directly present or cross-present the antigens to T cells, thereby amplifying the adaptive immune response.

Viral infections are prevented and cleared by both antibody-mediated humoral immunity and CD8+ T cell-mediated cellular immunity [49,50,51]. In this study, the humoral immune response was assessed by measuring serum levels of specific antibodies and cytokines (IFN-α and IL-4), while the cellular immune response was evaluated based on lymphocyte proliferation and IFN-γ levels. To assess immunological activity, mice were immunized with equivalent amounts of Bac-RP and Bac-p30. Both Bac-RP and Bac-p30 elicited humoral and cellular immune responses, with IgG antibody levels increasing over time (Figure 5B). Furthermore, compared with Bac-WT, Bac-RP and Bac-p30 induced higher expression of IFN-α (Figure 5E) and IL-4 (Figure 5G), indicating a robust humoral immune response in mice. The increase in IFN-α may be due to the adjuvant properties of the baculovirus vector, which enhances humoral and CTL responses by promoting dendritic cell maturation and inflammatory mediator production. However, given the intrinsic immunostimulatory activity of baculovirus DNA, these responses likely reflect non-specific innate immune activation rather than ASFV-specific effects. Further studies in porcine models are required to determine antigen-specific cytokine responses.

Cellular immune responses reportedly play a crucial role in protective immunity against intracellular ASFV infections [52]. IFN-γ secretion appears to contribute to this protective effect by activating macrophages, enhancing NK cell cytotoxicity, and initiating the transcription of target genes through the Janus kinase-signal transducer and activator of transcription (JAK-STAT) and nuclear factor kappa-B (NF-κB) signaling pathways [53,54]. A study using a baculovirus-expressed B602L-Fc fusion protein demonstrated enhanced immunogenicity of the African swine fever virus (ASFV) B602L protein. Mouse experiments showed that the B602L-Fc fusion protein effectively stimulated specific T lymphocyte proliferation and intracellular IL-4 and IFN-γ cytokine release [55]. To prevent interference with linear epitope display, Fc proteins were co-expressed. Compared to the Bac-WT control, Bac-RP induced higher IFN-γ levels (Figure 5D), consistent with previous studies. Furthermore, Bac-RP elicited strong T-cell responses, as indicated by increased CD4+ and CD8+ T-cell populations (Figure 6). These findings suggest that Bac-RP effectively induces both humoral and cellular immune responses. IFN-α and IL-4 correlate with humoral immunity, while IFN-γ is associated with cellular immunity, promoting Th1 cell differentiation.

Previous studies have shown that ASFV subunit vaccines reduce viral titers, prompting investigations into the functionality and epitopes of ASFV proteins [56,57]. Epitopes, the primary targets of antibodies or immune cells, can induce protective responses without requiring the entire antigen, thereby enhancing safety. Lu et al. identified an immunodominant epitope in the CD2v protein that is highly conserved across ASFV genotypes and elicits both humoral and cellular immune responses [58]. In a separate study, the four critical loops (ER1-4) of the ASFV p72 protein were individually fused to hepatitis B virus core particles (HBc), facilitating their self-assembly into nanoparticles. The immunogen 4G8 monoclonal antibody neutralized ASFV-positive sera by up to 84%, while a neutralization assay showed a 67% inhibition rate [59]. Inducing ASFV-specific humoral and cellular immune responses is essential for antiviral activity. Zhang et al. constructed three OprI fusion proteins, each incorporating epitopes from two different ASFV proteins, fused to the C-terminus of OprI and the N-terminus of the tetanus toxoid CD4+ T cell epitope. These results demonstrated that OprI fusion proteins activated dendritic cells and increased pro-inflammatory cytokine secretion. Furthermore, sera and peripheral blood mononuclear cells from vaccinated pigs reduced ASFV infection in vitro by 82.8% and 92.6%, respectively [60]. Epitope peptides that trigger immune responses and exhibit potential adjuvant activity are increasingly used in vaccine development due to their ability to modulate adaptive immune activation. They can be mass-produced via automated synthesis for short peptides and recombinant methods for longer epitopes and proteins. Epitope-based vaccines have been reported for several viruses, including SARS-CoV [61], SARS-CoV-2 [62], Lymphocytic Choriomeningitis Virus (LCMV) [63], Vesicular Stomatitis Virus (VSV) [64], and Human Papillomavirus (HPV) [65]. The identification of ASFV epitopes and the development of epitope-based peptide vaccines remain in the early stages, with many structural proteins and epitopes yet to be characterized. Advances in structural biology have enabled the structural analysis of ASFV protein antigens and the identification of antigenic epitopes [66,67,68,69]. Optimizing protective antigens through genomics and proteomics research can facilitate the design of safe and effective vaccines. Further insights into the mechanisms of protection and the antigens that elicit protective immunity are essential to determine which immune pathways confer robust and long-lasting protection and which antigen combinations enhance immune responses. Additionally, optimizing adjuvant selection, vaccination routes, dosage, pig breeds, and protective efficacy against homologous and heterologous strains is crucial for advancing vaccine development. Currently, epitope-based vaccines offer a promising strategy for subunit vaccine development. In this study, epitope proteins were engineered using baculovirus as a vector to enhance their immunogenicity. Their antiviral efficacy was improved by stimulating both cellular and humoral immune responses. In this study, epitope proteins were engineered using baculovirus as a vector with the aim of enhancing immunogenicity. The candidate stimulated both cellular and humoral immune responses in mice, which are associated with antiviral efficacy. Expanding the repertoire of ASFV epitopes for recombinant protein production represents a rationally designed strategy for vaccine development. A key limitation is the exclusive evaluation in mice, necessitated by the lack of BSL-3 facilities; consequently, any potential protective efficacy remains to be established in swine models through future challenge studies. Developing an ASF vaccine remains a complex challenge. In this study, the immune responses induced by Bac-RP were characterized in detail. Future studies must assess their safety and efficacy in pigs. However, the successful development of ASF vaccines depends on multiple factors, including immunogen selection, vaccine dosage and administration route, and the duration of protective immunity.

In this study, Bac-RP still needs to overcome certain limitations before it can be effectively developed as a vaccine for ASF control. Epitope proteins may offer a cost-effective alternative to cocktail formulations and represent a promising direction for future vaccine development. However, this approach requires a deeper understanding of ASFV immunological mechanisms.

## 5. Conclusions

In summary, the baculovirus-vectored construct functioned as a flexible antigen-delivery platform that elicited detectable immune responses in mice and could be evaluated further for use in a prime-boost vaccination strategy. The fusion expression of surface display signals enhances antigen uptake and presentation, effectively inducing humoral and cellular immune responses in mice. This approach provides a theoretical and technical foundation for developing live vector vaccines against ASFV.

## Figures and Tables

**Table 1:** List of primers for plasmid construction.

Primer Number	Primer Name	Sequence 5′-3′
1	RP-F	CTCGAGATGGGTCTGACCCGTACCA
2	RP-R	GCTAGCGTGGTGGTGGTGGTGGTGGCTATCTTTCGGCACTTCGC
3	p30-F	CTCGAGATGGATTTTATTTTAAATATATCCATGAAAATGGAGGTCATC
4	p30-R	GGTACCTTATTTTTTTTTTAAAAGTTTAAT
5	Actin-F	ACTGCCGAGCGTGAGATTGTC
6	Actin-R	GATGAAGGAGGGCTGGAACA
7	qRP-F	CGGTTTATTTGTCGCACTGCCTTTC
8	qRP-R	ATCAGAATCCGCATCAGCATCGT
9	q30-F	ATGAATGTTCTCCGAAGATGCTG
10	q30-R	ATGATATTGTGAAATCTGCTCGT
11	Gp-64-F	CGGCGTGAGTATGATTCTCAAA
12	Gp-64-R	ATGAGCAGACACGCAGCTTTT

## Data Availability

The original contributions presented in this study are included in the article. Further inquiries can be directed to the corresponding authors.
